# Initiation codon mutation in βB1-crystallin (*CRYBB1*) associated with autosomal recessive nuclear pulverulent cataract

**Published:** 2009-05-18

**Authors:** Esther Meyer, Fatimah Rahman, Jessica Owens, Shanaz Pasha, Neil V. Morgan, Richard C. Trembath, Edwin M. Stone, Anthony T. Moore, Eamonn R. Maher

**Affiliations:** 1 Department of Medical and Molecular Genetics, Institute of Biomedical Research, University of Birmingham, Birmingham, UK; 2 The Carver Family Center for Macular Degeneration, University of Iowa, Molecular Ophthalmology Laboratory, Iowa City, IA; 3 Division of Medical Genetics, Departments of Medicine and Genetics, University of Leicester, Leicester, UK; 4 Department of Ophthalmology & Visual Science, University of Iowa, Iowa City, IA; 5 Institute of Ophthalmology, University College London, London, UK

## Abstract

**Purpose:**

To identify the molecular basis for autosomal recessively inherited congenital non-syndromic pulverulent cataracts in a consanguineous family with four affected children.

**Methods:**

An autozygosity mapping strategy using high density SNP microarrays and microsatellite markers was employed to detect regions of homozygosity. Subsequently good candidate genes were screened for mutations by direct sequencing.

**Results:**

The SNP microarray data demonstrated a 24.96 Mb region of homozygosity at 22q11.21-22q13.2 which was confirmed by microsatellite marker analysis. The candidate target region contained the β-crystallin gene cluster and direct sequencing in affected family members revealed a novel mutation in *CRYBB1* (c.2T>A; p.Met1Lys).

**Conclusions:**

To our knowledge this is the first case of an initiation codon mutation in a human crystallin gene, and only the second report of a *CRYBB1* mutation associated with autosomal recessive congenital cataracts. In addition, although a number of genetic causes of autosomal dominant pulverulent cataracts have been identified (including *CRYBB1*) this is the first gene to have been implicated in autosomal recessive nuclear pulverulent cataract.

## INTRODUCTION

Congenital cataract is a major cause of visual loss in children worldwide with an estimated incidence of about 1 per 4,000 live births [1]. This disease has multiple causes; however genetic factors play an important role in its etiology. Cataract can be inherited as an isolated trait, in association with other ocular anomalies, or as part of systemic syndromes. The majority of isolated congenital cataracts show autosomal dominant inheritance, but autosomal recessive and X-linked forms have also been observed [2].

Inherited cataracts demonstrate extreme genetic heterogeneity, with more than 20 genes identified to date. About half of reported inherited cataract mutations are in crystallin genes [3]. Crystallins constitute about 90% of the water-soluble proteins of the lens and are divided into two major classes, the α-crystallin family and the β/γ-crystallin superfamily. The α-crystallins are heat shock proteins that function as molecular chaperones. The β- and γ-crystallins share a common structural feature consisted of four “Greek key” motifs. The major sequence difference between oligomeric β-crystallins and monomeric γ-crystallins is that β-crystallins have long terminal extensions [4]. To date mutations in 10 human crystallin genes have been associated with inherited cataracts [5]. Of these, mutations in 9 crystallin genes have been associated with autosomal dominant cataracts (*CRYAA*, *CRYAB*, *CRYBB1*, *CRYBB2*, *CRYBA1/A3*, *CRYBA4*, *CRYGC*, *CRYGD*, and *CRYGS*) but only 3 with autosomal recessive cataracts (*CRYAA*, *CRYBB1*, and *CRYBB3*). In order to further delineate the molecular pathology of autosomal recessive cataracts, we investigated a consanguineous family with nuclear pulverulent cataracts and identified a novel germline *CRYBB1* mutation.

## METHODS

### Patients

A consanguineous family of Somali origin with four affected children was ascertained and recruited for molecular genetic analysis. All subjects gave written informed consent. The study was approved by the South Birmingham Local Research Ethics Committee and was performed in accordance with the Declaration of Helsinki.

### Molecular genetic studies

Genomic DNA from the four affected individuals, two unaffected siblings, and the mother were extracted from peripheral blood samples by standard techniques. For linkage studies a genome-wide linkage scan was carried out in the affected individuals using Affymetrix 250k SNP microarrays (Affymetrix Pte Ltd, Singapore). DNA was amplified and hybridized to the Affymetrix SNP chip according to manufacturer’s instruction. Candidate regions of homozygosity (>2 Mb) were evaluated by typing microsatellite markers in all family members. Information on primers and the physical order of the markers was obtained from the NCBI database and from the UCSC browser, respectively. Amplification conditions were an initial denaturation of 94 ºC for 3 min, followed by 28 cycles of 30 s denaturation at 94 ºC, 30 s annealing at 55 ºC, and 30 s extension at 72 ºC with a final extension at 72 ºC for 5 min. The alleles were detected by an automated ABI 3730 DNA Analyzer and product sizes were determined using Genemapper v3.0 software (Applied Biosystems Inc., Foster City, CA). Mutation analysis of *CRYBB1*, *CRYBB2*, *CRYBB3*, and *CRYBA4* was undertaken by direct sequencing. The genomic DNA sequence of these genes was taken from Ensembl and primer pairs for the translated exons were designed using primer3 software. Amplification was performed according to standard protocols with *Taq* polymerase provided by ABgene (Abgene Limited, Epsom, United Kingdom). PCR products were directly sequenced by the Big Dye Terminator Cycle Sequencing System with the use of an ABI PRISM 3730 DNA Analyzer (Applied Biosystems Inc.). DNA sequences were analyzed using Chromas software.

## RESULTS

### Clinical Findings

**Figure 1:**
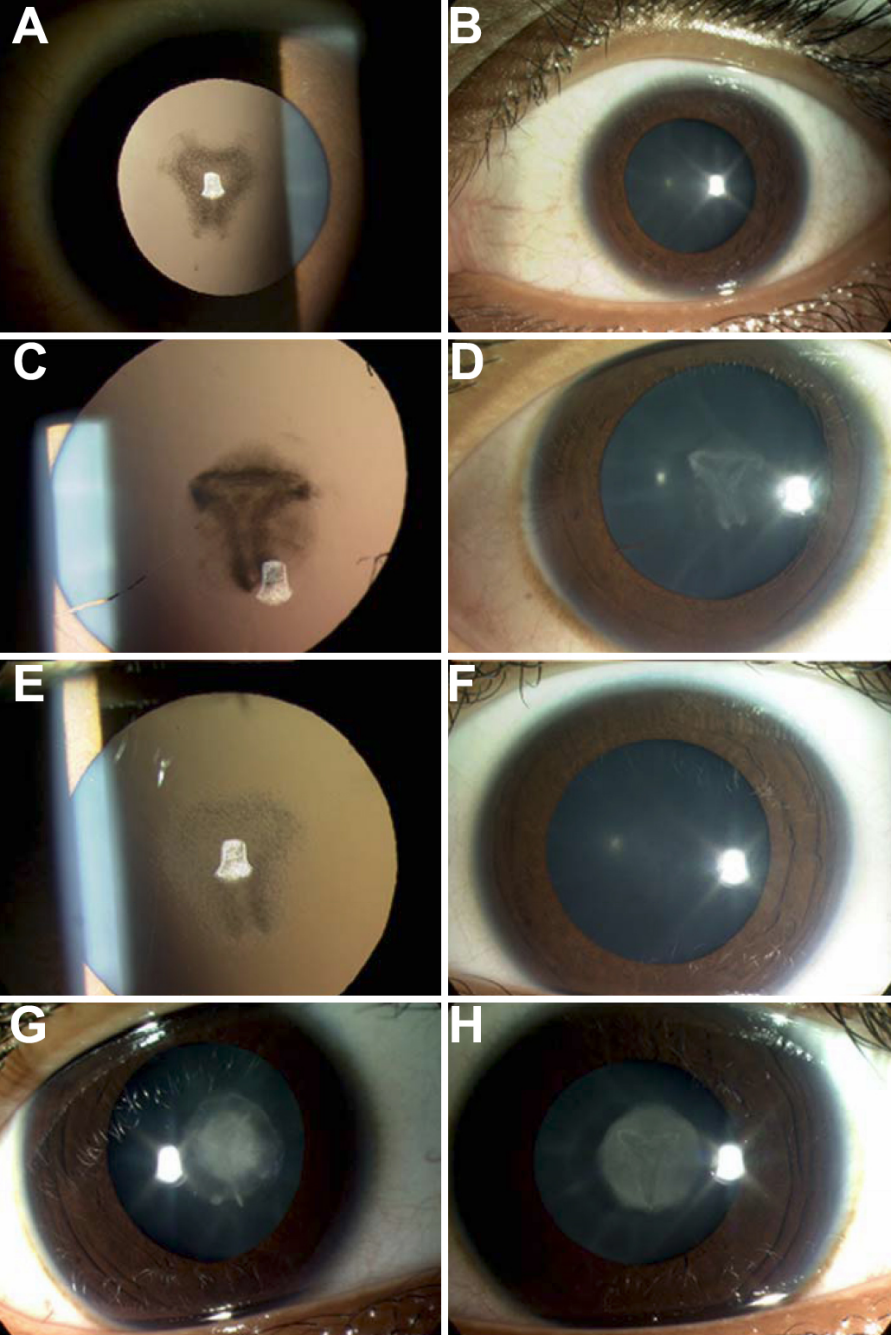
Cataract phenotype of family. **A**: Left eye retroillumination view and **B**: left eye slitlamp view of patient II:3. **C**: Left eye retroillumination view and **D**: left eye slit lamp view of patient II:5. **E**: Left eye retroillumination view and **F**: left eye slit lamp view of patient II:6. **G**: Right eye slit lamp view and **H**: left eye slit lamp view of patient II:4.

Clinical examination of all affected family members revealed nuclear cataract in both eyes. The rest of the eye examination was normal. The milder affected individuals (II:3, II:5, and II:6) had obvious pulverulent opacities whereas II:4 had a dense nuclear opacity without obvious pulverulent changes (Figure 1).

### Genetic linkage studies

**Figure 2:**
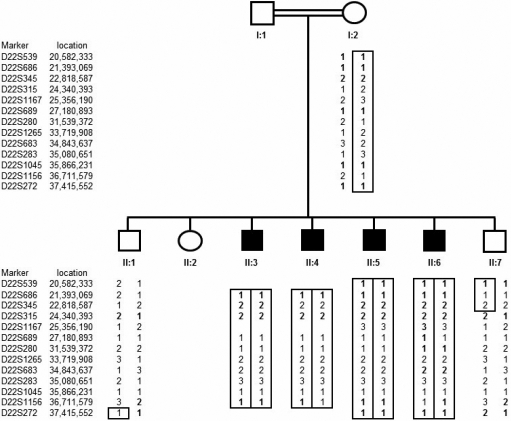
Cataract pedigree and haplotype analysis. Pedigree and haplotype analysis of Somali cataract family shows segregation of microsatellite markers surrounding β-crystallin cluster on chromosome 22.

Genome-wide genotyping using the Affymetrix 250k SNP microarrays in affected individuals II:5 and II:6 (Figure 2) revealed seven extended regions of homozygosity; 13.96 Mb and 12.02 Mb on chromosome 3 (from 8.69 to 22.65 Mb and from 167.34 to 172.75 Mb), 44.72 Mb on chromosome 8 (from 57.82 to 102.53 Mb), 4.77 Mb on chromosome 10 (from telomere to 4.77 Mb), 20.35 Mb on chromosome 15 (from 25.47 to 45.82 Mb), 6.14 Mb on chromosome 21 (from 30.29 to 33.28 Mb), and 24.96 Mb on chromosome 22 (from 16.96 to 41.92 Mb). Linkage to known cataract genes and loci could be excluded except for *EYA1* (72.27 to 72.44 Mb) on chromosome 8 and the β-crystallin gene cluster on chromosome 22. Further genotyping was then undertaken in all available family members (mother [I:2], two unaffected siblings [II:1 and II:7], and four affected individuals [II:3, II:4, II:5, and II:6]) using microsatellite markers for these regions. Whilst one of the affected individuals (II:4) was heterozygous for each microsatellite marker on chromosome 8 (results not shown), all affected individuals had a identical homozygous haplotype on chromosome 22. Furthermore the microsatellite marker analysis indicated that the two unaffected siblings had a different haplotype due to the inheritance of the opposite maternal allele (Figure 2). Multipoint linkage analysis gave a maximum LOD score of 3.14 at D22S683. These findings were consistent with linkage to 22q11.21-q13.2.

### Mutation analysis of candidate genes

**Figure 3:**
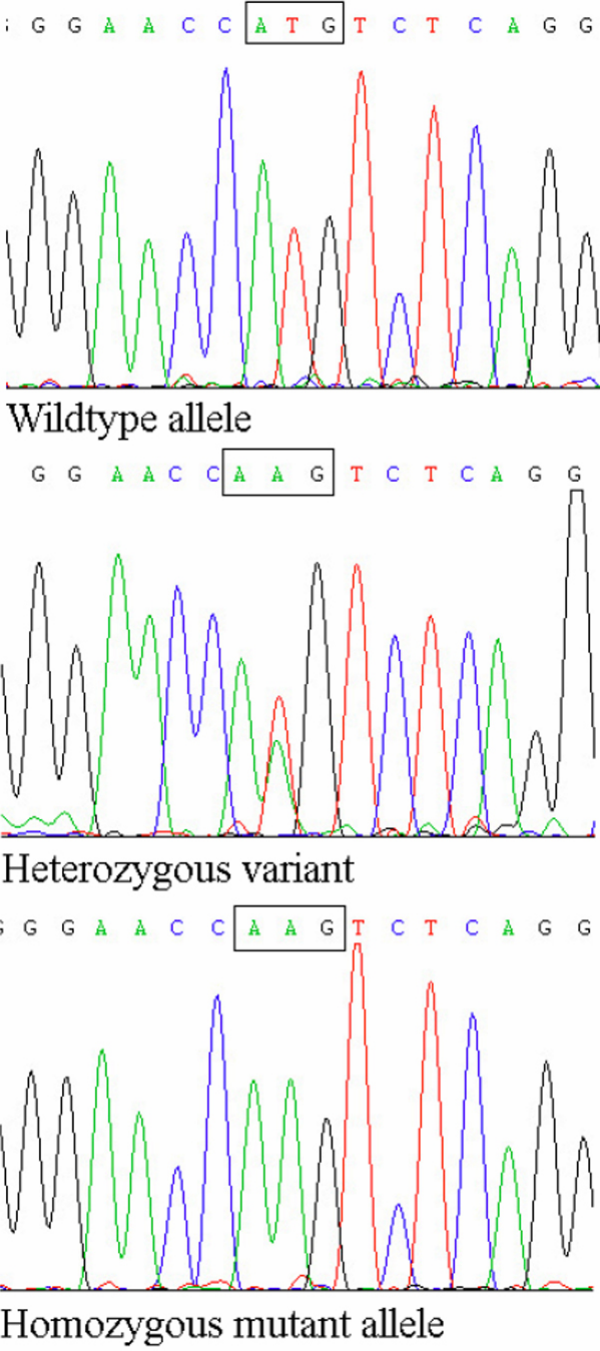
*CRYBB1* mutation. In top row is the wildtype sequence in a control; in the middle row is a heterozygous *CRYBB1* mutation (c.2T>A) in the mother; and at the bottom is a homozygous *CRYBB1* variant (c.2T>A) in an affected individual.

The 24.96 Mb candidate interval at chromosome 22q11.21-q13.2 contained 644 known genes. Significantly a cluster of four genes encoding members of the β-crystallin family were contained within the target interval (*CRYBB1* at 25.33 Mb, *CRYBB2* at 23.95 Mb, *CRYBB3* at 23.93 Mb, and *CRYBA4* at 25.35 Mb). Hence direct sequencing of *CRYBB1*, *CRYBB2*, *CRYBB3*, and *CRYBA4* was then carried out. Mutation analysis of *CRYBB2*, *CRYBB3*, and *CRYBA4* showed no evidence of a pathogenic mutation. However a homozygous T→A substitution in the ATG initiation codon of the *CRYBB1* gene (c.2T>A; p.Met1Lys) was identified (Figure 3). This sequence variant cosegregates with the disease phenotype and was found to be homozygous in all affected individuals and heterozygous in the mother. The mutation was not detected in 242 control Somali chromosomes or in 262 general laboratory control chromosomes.

## DISCUSSION

We identified an initiation codon mutation in *CRYBB1* in a family with autosomal recessive form of congenital cataract (nuclear pulverulent cataract). Mutations affecting the initiation codon have been described in a wide range of human disease genes [6]. To our knowledge, this is the first report of an initiation codon mutation in a human crystallin gene (although an initiation codon mutation has been described in a murine cataract model – see later). In mammals an AUG codon is optimum for translational initiation, nevertheless the efficiency of translation initiation is also influenced by the surrounding sequence (in particular a purine at position -3 and G at position +4) [7]. However some non-AUG triplets are able to direct translation initiation, this does not include AGG and AAG [8]. In our family the mutation produced an AAG codon which is not competent to initiate translation. In such circumstances translation might start at a downstream AUG or the mutation may produce a null allele [9]. In *CRYBB1* the next downstream AUG is codon 113 but the flanking sequence is not optimal for translation initiation (GGGGAGaugU). Even if translation was initiated from this codon, the translated protein would lack the first 112 amino acids of the wild type protein including the Greek key I and part of the Greek key II motif (UniProtKB/Swiss-Prot P53674). However, similar initiation codon mutations have been reported in patients with β-thalassemia [10] and triose phosphate isomerase deficiency [11] and were thought to result in a null allele. Furthermore Graw et al. [12] identified an initiation codon mutation in a murine crystallin gene causing a phenotype with nuclear and zonular cataract. This ethylnitrosourea-induced mutation disrupted the start codon of the *Cryge* gene altering the ATG to a TTG codon.

To our knowledge, there are only five previous reports of *CRYBB1* mutations in patients with congenital cataract and only one of these in a patient with autosomal recessive cataract (Table 1). Interestingly, all sequence changes reported in autosomal dominantly inherited cataract families are located in exon 6, which encodes the Greek key IV and the COOH-terminal extension [4,13-15]. These mutations might be predicted to result in an abnormally elongated or truncated COOH-terminus and production of a mutant protein. In contrast the two mutations associated with autosomal recessive cataracts both occurred in exon 2. These more 5’ mutations might be predicted to lead to an absence of functional protein product either by abrogation of translation or by nonsense mediated mRNA decay (as suggested for the N58Tfs106 mutation reported by Cohen et al. [16]). Thus it can be postulated that mutations that result in the production of an abnormal protein will have a dominant negative effect and cause dominantly inherited cataracts whilst mutations associated with loss of protein expression will cause recessive cataracts. Consistent with this hypothesis the mother in our family who was heterozygous for the loss of function translation initiation codon mutation did not have any evidence of a cataract.

**Table 1:** Known mutations in *CRYBB1* in association with isolated congenital cataract.

**Inheritance**	**Phenotype**	**Mutation: protein-level**	**Exon**	**Reference**
Autosomal dominant	bilateral, pulverulent, affected fetal nucleus, cortex and anterior and posterior Y-suture region	G220X → truncated βB1-crystallin → partial loss of 4. Greek-key motif	6	[13]
Autosomal dominant	dense nuclear with cortical riders and anterior and posterior polar opacities and microcornea	X253R → elongation of COOH-terminus → disruption of β-crystallin interactions	6	[4]
Autosomal dominant	bilateral nuclear cataract	S228P → disturb consecutive β-sheet and make a β-turn	6	[14]
Autosomal dominant	bilateral, disc-like opacities in central nucleus region	Q223X → truncated βB1-crystallin → partial loss of 4. Greek-key motif	6	[15]
Autosomal recessive	bilateral confluent nuclear opacification	N58Tfs106 → abrogates protein very near to NH_2_-terminus	2	[16]
Autosomal recessive	bilateral, mild nuclear pulverulent cataract	M1K → abrogates initiation codon	2	current study

Congenital cataracts are genetically and phenotypically heterogeneous. The relationships between genotype and phenotype are complex as a specific clinical cataract phenotype may be seen in association with mutations in several different genes and mutations in a single gene can result in a variety of cataract phenotypes [2]. The severity of cataract can vary within families as seen in our cases but the type of cataract usually remains constant [17]. The phenotype on our family is consistent with a nuclear pulverulent cataract. Three of the four affected individuals had the distinctive powdery lens opacities seen in this type of cataract while the more severely affected individual had dense nuclear cataracts. Such variation in the severity is common in pulverulent cataract [17].

*CRYBB1* mutations have been associated with a number of cataract subtypes (e.g. pulverulent and nuclear) and with additional developmental ocular abnormalities (microcornea; Table 1). Mutations in a number of genes have been identified for autosomal dominant pulverulent cataracts including (*CRYGC*, *CRYBA1*, *CRYBB1*, connexin46/*GJA3*, connexin50/*GJA8*, and *VIM*) [13,18-30]. Although a locus for late onset pulverulent cataracts was mapped previously to 9q13-q22 [31], our findings implicate *CRYBB1* as the first gene to be associated with autosomal recessive nuclear pulverulent cataracts. Further characterization of autosomal recessive forms of inherited cataracts will expand knowledge of cataractogenesis and provide a basis for genotype-phenotype studies that can provide insights into gene function and disease pathogenesis.
